# Relationship between headache and Internet addiction in children

**DOI:** 10.3906/sag-1806-118

**Published:** 2019-10-24

**Authors:** İlknur TEPECİK BÖYÜKBAŞ, Ayşegül Neşe ÇITAK KURT, Selma TURAL HESAPÇIOĞLU, Mehmet UĞURLU

**Affiliations:** 1 Department of Family Medicine, Azdavay State Hospital, Kastamonu Turkey; 2 Department of Pediatric Neurology, Faculty of Medicine, Yıldırım Beyazıt University, Ankara Turkey; 3 Department of Child and Adolescent Psychiatry, Faculty of Medicine, Yıldırım Beyazıt University, Ankara Turkey; 4 Department of Family Medicine, Faculty of Medicine, Yıldırım Beyazıt University, Ankara Turkey

**Keywords:** Child, headache, Internet addiction, migraine, prevalence

## Abstract

**Background/aim:**

We aimed to investigate Internet addiction in pediatric patients with migraine- and tension-type headache in this study.

**Materials and methods:**

Among our 200 subjects, 103 had migraine-type headache and 97 had tension-type headache.

**Results:**

Headache triggered by computer use was more common in the migraine-type headache group. There was no difference between the Internet addiction scale score of the two groups. The Internet addiction scale scores of the patients were found to differ depending on the aim and duration of computer use. Internet addiction was found in six (6%) patients. Internet addiction prevalence was 3.7% and 8.5% in the two groups, respectively.

**Conclusion:**

The prevalence of Internet addiction in children with recurrent headache was lower than that found in their peers in Turkey, possibly due to avoidance of computer use as a headache trigger. This finding raises the question of whether migraine- or tension-type headaches actually prevent Internet addiction.

## 1. Introduction

Headache is as old as humanity and is common in both children and adults. Headache significantly affects the life quality, education, and play of children. Various environmental factors such as nutrition, insomnia, sun, stress, and computer use are as important as genetic factors in the occurrence of primary recurrent headaches and especially migraine-type headaches (MTH) in children [1].

As the use of computers and the Internet has become widespread worldwide, these technological tools have become an indispensable part of human life. Increased use of computers in Turkey has increased Internet access rates and decreased the age of initial Internet use. Problems that may be caused by the use of computers and the Internet have therefore also become as important in children as in adults. Internet use has provided easy access to information and facilitated business and scientific life, but has also increased sleep disorders, psychosocial problems, musculoskeletal problems, headaches, obesity, and Internet addiction due to mindless and excessive use [2–5].

Internet addiction has been defined by Young as the inability to prevent the excessive use of Internet with the time spent without Internet connection being deemed insignificant, the presence of extreme irritability and aggression when deprived, and the continuing deterioration of the work, social, and family life of the individual [6]. Adolescents are more predisposed to Internet addiction than adults. The incidence of Internet addiction in adolescents has been reported as 11% in China, 8% in Greece, and 18.4% in Korea. Internet addiction rates of 6.6%–15% have been reported in recent studies from Turkey [2,3].

Excessive use of the Internet is known to be related to psychiatric disorders such as depression, anxiety, sleep disorders, lack of attention, and hyperactivity as a result of negative psychosocial development in adolescents [7,8]. However, only a few studies have investigated the opposite hypothesis: whether the presence of Internet addiction is affected in patients with headache [9].

Cerutti et al. [9] conducted a population-based study to investigate whether adolescents with migraine-type (MTH) or tension-type headache (TTH) showed higher rates of Internet and/or mobile phone abuse. They found no difference between the children with and without headache in terms of Internet or phone abuse. There was also no difference between the MTH and TTH groups in terms of media abuse. They reported no difference in terms of headache when the subjects were divided into 4 groups, i.e. no addiction, only Internet addiction, only phone addiction, and both Internet and phone addiction. However, they obtained their data from self-reporting scales. They mentioned the limitations of self-reporting scales themselves in the study. The subjects were not interviewed face-to-face.

Our aim was to investigate the characteristics of Internet use and addiction in child patients with migraine- or tension-type headache when the cases of a clinical sample were evaluated face-to-face by a pediatric neurologist as well as a child and adolescent psychiatrist. We therefore aimed to contribute to the literature regarding Internet use habits and Internet addiction rates of children with MTH and TTH. 

## 2. Materials and methods

The study was conducted at Ankara Yıldırım Beyazıt University Yenimahalle Training and Research Hospital’s Department of Child Neurology and Child and Adolescent Mental Health Department. 

Approval was obtained from Ankara Yıldırım Beyazıt University Yenimahalle Training and Research Hospital’s Clinical Studies Ethics Committee.

### 2.1. The selection of the study groups

Patients who presented to the above clinics with symptoms of headache and who were diagnosed with MTH or TTH based on the International Classification of Headache Disorders 3 beta (ICHD 3β) criteria [10], agreed to participate in the study, and met the criteria below were included in the study. 

Study inclusion criteria:

**- **Diagnosed with MTH in accordance with ICHD 3β criteria;

-****Diagnosed with TTH in accordance with ICHD 3β criteria; 

- Without additional neurologic and/or systemic disease.

Study exclusion criteria:

-****Diagnosis other than MTH in accordance with ICHD 3β criteria; 

- Any additional neurologic and/or systemic disease.

A total of 200 consecutive patients who presented to the outpatient clinic with headache and were diagnosed with MTH or GTH were included in the study in accordance with study inclusion criteria. We also administered the Internet Addiction Scale (IAS) to 100 patients who reported daily Internet usage on questioning and were above the age of 12 years. 

### 2.2. Data collection tools

#### 2.2.1. Sociodemographic and clinical data form

The sociodemographic characteristics of the cases such as age, gender, and educational status of the parents as well as characteristics of the headache, headache triggering factors, presence of a computer and Internet at home, and characteristics of Internet use (purpose of use, daily duration of Internet use) were included in the form developed by the investigators. 

#### 2.2.2. Internet Addiction Scale (IAS)

The Turkish validity and reliability study of this scale developed by Nichols and Nicky [4] has been conducted in patients above the age of 12 [5]. IAS is completed by the patient as a self-reported scale and consists of 27 items with 5 options. The options are “never” (1 point), “rarely” (2 points), “sometimes” (3 points), “often” (4 points), and “always” (5 points). After IAS administration was completed, the points were summed up and the IAS score calculated. We classified 6 patients with an IAS score over 81 as having Internet addiction (IA). These subjects were evaluated by the Child and Adolescent Mental Health Department (STH), and the necessary follow-up and treatment were started.

### 2.3. Statistical evaluation methods

Statistical evaluation was conducted with the SPSS (version 21.0, SPSS Inc., Chicago, IL, USA) program. Nonparametric (Kruskal–Wallis, chi-square test) and parametric (Student’s t-test) tests were used for the comparisons between the groups. The chi-square test was used in the comparison of headache triggering factors and gender of the patients with MTH and TTH. When the general characteristics of the group and daily duration of Internet use were compared, the Kolmogorov–Smirnov test was used first, followed by Student’s t-test for data consistent with a normal distribution and the Mann–Whitney U test for other data. The t or Z values are presented based on the analysis used. Spearman correlation was used for correlation analyses. The Kruskal–Wallis test was used for the comparison of mean IAS scores based on the duration and most common purposes of Internet use. Post hoc comparisons were conducted with the Scheffe test. A P value of <0.05 was accepted as significant in the entire study.

## 3. Results

Of the 200 patients evaluated, 103 had MTH and 97 had TTH. The mean age was 151.6 ± 37.6 months and the F/M ratio 65/38 in the MTH patients. These figures were 142.8 ± 34.3 months and 54/43, respectively, for the TTH group, with no statistically significant difference between the groups (P = 0.315, X2 = 1.146). Headache triggering factors were present in 99 (96.1%) patients in the MTH group and 78 (80.4%) patients in the TTH group (Table 1).

**Table 1:** Headache triggers of the patients.

	MTBA (n, %)	GTBA (n, %)		
Headache triggers			P	X2
Physical activity	87 (84.5)	50 (51.5)	<0.0001*	25.089
Stress, sadness	84 (81.6)	61 (62.9)	0.004*	8.731
Hunger	73 (70.9)	44 (45.4)	<0.0001*	13.394
Insomnia	70 (68.0)	57 (58.8)	0.189	1.824
Sunlight	62 (60.2)	43 (44.3)	0.033*	5.042
Computer use	59 (57.3)	39 (40.2)	0.017*	5.828
Watching television	57 (55.3)	35 (36.1)	0.007*	7.458
Certain foods	21 (20.4)	7 (7.2)	0.008*	7.199

195 (97.5%) patients had a computer at home and 182 (91%) had an Internet connection at home. The Internet was used daily by 180 (90%) patients; the most common reasons were games and social network use (Table 2).

**Table 2:** The reason for Internet use in MTH and TTH patients.

Purpose of Internet use*	MTH	TTH	P	X2
Game	26	41	0.011	6.500
Social network use	42	32	0.254	1.300
Preparing homework	28	17	0.102	2.673
Movies, music	15	20	0.260	1.269

*The patients were allowed to report multiple reasons.

 The daily duration of Internet use was 2.3 ± 1.7 h. The majority used the Internet for more than 1 h daily. Daily duration of Internet use was 1.95 ± 1.67 h in the MTH group and 2.22 ± 1.90 h in TTH group, with no statistically significant difference between the groups (P =0.212, Z = –1.248). The mean IAS score of the 100 patients using the Internet daily and who were above the age of 12 was 51.2 ± 17.9. Although the IAS score was higher in females, this difference was not statistically significant (P > 0.05)

When IAS scores were compared according to the most common aim of Internet use, a statistically significant difference was found between the groups (P = 0.017, Table 3). The IAS scores of those using social networks were significantly higher than the scores of those who were using the Internet for games (P = 0.033, Table 3), but no statistically significant difference was found between the other groups with post hoc analysis.

**Table 3:** Patient characteristics of Internet use (patients administered IAS) and IAS scores.

	To t a l(n: 100)	IAS score(mean ± SD)
The purpose of Internet use		
Social networks	46	54.9 ± 19.6*
Movies, music	24	49.2 ± 16.5
Preparing homework	16	52.2 ± 14.0
Game	14	41.7 ± 15.9*
Daily duration of Internet use		
Less than 1 h	29	43.4 ± 13.7*
Between 1–2 h	20	53.0 ± 18.5
More than 2 h	51	55.0 ± 18.7*

*: P < 0.05.

When the patients were grouped based on the duration of Internet use, a statistically significant difference was found between the IAS scores of the three groups (P = 0.005). The IAS scores of those using the Internet for less than 1 h daily were significantly lower than the scores of those who were using it for more than 2 h (P = 0.005), but no statistically significant difference was found between the other groups for IAS score in the post hoc analysis (Table 3). 

A statistically significant positive correlation was present between the duration of daily Internet use and IAS scores (P = 0.006; r = 0.272).

When the educational level of the parents was evaluated based on the adolescent’s IAS scores, adolescents with high school graduate parents were found to have the highest scores, but this difference was not statistically significant (educational level of the mother P = 0.743, educational level of the father P = 0.125, Table 4). No significant correlation was present between educational level of the parents and IAS scores of the children (P = 770, r = –0.30, P = 0.608, r = 0.052, respectively).

**Table 4:** Educational level of the parents and IAS distribution.

Educational level	Total(n: 100)	IASmean ± SD
Educational level of the mother		
Primary school	30	51.8 ± 18.6
Secondary school	12	47.9 ± 16.2
High school	34	53.3 ± 19.3
University	24	49.3 ± 16.2
Educational level of the father		
Primary school	20	49.5 ± 19.5
Secondary school	15	46.1 ± 14.2
High school	45	54.2 ± 18.8
University	20	50.2 ± 16.8

Of the 180 patients reporting daily Internet use, 53 of 100 patients who were administered IAS tests were in the MTH group and 47 in the TTH group. The IAS score was 52.4 ± 15.4 in the MTH group and 49.9 ± 20.4 in the TTH group. No difference was found between the groups in terms of the IAS score (P > 0.05). There were 6 (6%) patients found to have Internet addiction with an IAS score over 81; 2 were in the MTH group and 4 in the TTH group. All of the patients who were found to have IA were female. The Internet addiction prevalence was 3.7% in the MTH group and 8.5% in the TTH group.

## 4. Discussion

We investigated the characteristics of Internet use and Internet addiction in pediatric patients with migraine- or tension-type headache in this study. Headache significantly decreases the life quality of adults and is also one of the most common reasons for school absenteeism in children. The symptoms have been found to start in childhood in the history of migraine patients diagnosed in adulthood [11].

The most important causes of the most common recurrent primary headaches in children are MTH and TTH. The reported MTH and TTH prevalence in children varies in different studies [12,13]. A study from our country has reported a primary recurrent headache prevalence of 21%, MTBA prevalence of 7.2%, and GTBA prevalence of 7.8% in children aged 7–17 years [12]. MTBA is reported to be more common in girls [13,14]. The F/M ratio in our study was 65/38 in the MTH group and 54/43 in the TTH group. All of our cases who presented consecutively as pediatric neurology outpatients agreed to participate and were included in the study; thus, MTH and TTH can both be said to be more common in girls. 

Triggers are quite important in headaches and especially in migraine. Migraine attacks can be prevented with the detection and elimination of the triggering factor in certain patients [13,14]. Hunger, physical activity, stress, sadness, watching television, computer use, and certain foods were the most common headache triggers in our MTH group. 

There was a computer at home for 97.5% of our patients; 91% also had a home Internet connection. The Internet was used daily by 90% of this group and the most common reasons were games and social networks. A study conducted in an older age group found that the Internet was used most commonly for social networks and communication, while the number of those using it for games was low [3]. This indicates that the reason for Internet use varies based on the age of the user.

Spending long periods of time on the computer has been reported to be one of the primary recurrent headache triggers in children in recent years [13]. Headache was said to be triggered by computer use in 57.3% of the patients in our MTH group and 40.2% in the TTH group. Computer and Internet use has been increasing rapidly in our country and around the world in recent years. Although it is mostly seen as a device for the education and entertainment of children, uncontrolled and uninformed use can cause serious problems. These problems can present as headache, fatigue, sleep problems, dizziness, neck and back pain, memory and learning difficulties, social isolation, difficulty in self-expression, depression, anxiety, and Internet addiction [15,16]. Children are more sensitive to the negative effects of Internet use than adults [9]. The TTH group reported spending significantly more time playing computer games in our study than the MTH group. The time spent using the Internet was also higher in the TTH group. Our patients with MTH reported significantly higher rates of headache triggered by computer use. This can explain the shorter durations for which children and adolescents with MTH use a computer. 

IAS was administered to 100 patients above the age of 12 among the 200 patients in our study group. Although the IAS scores of female patients were higher than those of the male patients, this difference was not statistically significant. The Internet addiction rate was 9.3% in the girls and 20.4% in the boys in another study [3]. Canan et al. [5,17] found the Internet addiction risk of boys to be higher. This increased rate was related to male children playing Internet games more often. In contrast, some studies have reported the Internet addiction rate to be 7.6% in girls and 6.2% in boys [2]. The fact that all 6 patients with an IAS score over 81 in our study were female adds a different dimension to this condition in the literature.

Internet addiction prevalence was 3.7% in MTH patients and 8.5% in GTH patients above the age of 12 in our study. GTBA patients reported that they used the Internet more and played more games. Computer use was a significant trigger factor in migraine patients in our study. This demonstrates that adolescents with migraine use the computer less and that the Internet addiction prevalence is also less. 

The highest IAS addiction scores were found in those who used social networks most commonly. IAS score was higher in those using the Internet for homework than those using it for music. Canan et al. [5,17] found a higher risk of Internet addiction when the Internet was used for surfing. The group with the highest risk for Internet addiction used it for Internet surfing and social networks in another study [3]. Our study group had different reasons for Internet use compared to groups in other studies as the patients were younger. It is important to consider age groups and Internet use preferences of different age groups when investigating Internet addiction and pathological Internet use.

When the daily duration of Internet use and IAS scores were evaluated, more than 2 h was found to increase the IAS score. A significant linear correlation was present between the duration of Internet use and the IAS score. The Internet addiction risk increased significantly with a daily duration of Internet use over 2 h [9]. It is therefore important for families to monitor the aim and duration of Internet use by their children. Internet addiction rates were found to be higher in children of neglectful parents who did not supervise their children in studies investigating the relationship between family attitudes and Internet addiction in children [18].

When the IAS scores of children were evaluated according to the educational level of their parents, the scores of children with high school graduate parents was higher. Similarly, although the Internet addiction rate was found to be higher in those with high school graduate parents in another study, this was not statistically significant [3]. This can be explained with the increase in educational level leading to increased socioeconomic level and facilitating access to a computer and the Internet. Increased amount of time without parental supervision in the case of working parents may also have caused this situation.

Cerutti et al. [9] excluded students who were diagnosed with any physical or mental disease and were receiving pharmacotherapy. They may therefore have excluded individuals who were under follow-up and treatment with a diagnosis of headache. Our sample consisted of cases presenting to the Pediatric Neurology outpatient clinic with the symptoms of headache. Additionally, the diagnoses based on self-reporting scales in Cerutti et al.’s study may not have included clinically ill cases. We diagnosed migraine and tension-type headache with face-to-face interviews and evaluation by a pediatric neurology specialist based on the ICDH 3 beta version. Patients with high IAS values were also evaluated face-to-face by a child and adolescent mental health specialist, and IA presence was verified. 

The rate of Internet addiction in adolescents was found to be 11.6% in a previous study from Turkey [5]. We found an Internet addiction prevalence of 3.7% in MTH patients and 8.5% in TTH patients. This lower rate in our patients may be due to computer use being an important trigger for headache; therefore, there is less use of the computer in order to avoid headache attacks. The results of this study lead to the question of whether headaches in children, especially MTH, are a factor protecting adolescents from Internet addiction. Cerutti et al. [9] observed Internet addiction rates in a sample of students where cases diagnosed with a medical or mental disease or receiving pharmacotherapy were excluded. Longitudinal studies with larger samples are required to reach more definite conclusions on this issue.
